# Immunotopographical Differences of Human Skin

**DOI:** 10.3389/fimmu.2018.00424

**Published:** 2018-03-05

**Authors:** Gabriella Béke, Zsolt Dajnoki, Anikó Kapitány, Krisztián Gáspár, Barbara Medgyesi, Szilárd Póliska, Zoltán Hendrik, Zoltán Péter, Dániel Törőcsik, Tamás Bíró, Andrea Szegedi

**Affiliations:** ^1^Division of Dermatological Allergology, Department of Dermatology, Faculty of Medicine, University of Debrecen, Debrecen, Hungary; ^2^Department of Dermatology, Faculty of Medicine, University of Debrecen, Debrecen, Hungary; ^3^Genomic Medicine and Bioinformatic Core Facility, Department of Biochemistry and Molecular Biology, Faculty of Medicine, University of Debrecen, Debrecen, Hungary; ^4^Department of Pathology, Faculty of Medicine, University of Debrecen, Debrecen, Hungary; ^5^Department of Immunology, Faculty of Medicine, University of Debrecen, Debrecen, Hungary

**Keywords:** antimicrobial peptides, barrier function, chemokines, IL-17, sebaceous glands, skin, T cells

## Abstract

The immunological barrier of the healthy skin is considered to be unified on the whole body surface—however, recent indirect findings have challenged this dogma since microbial and chemical milieu (e.g., sebum, sweat, and pH) exhibit remarkable differences on topographically distinct skin areas. Therefore, in the present study, we performed whole transcriptomic and subsequent pathway analyses to assess differences between sebaceous gland rich (SGR) and sebaceous gland poor (SGP) regions. Here, we provide the first evidence that different skin regions exhibit a characteristic innate and adaptive immune and barrier milieu as we could detect significantly increased chemokine (CCL2, 3, 19, 20, 23, 24) and antimicrobial peptide (S100A7, A8, A9, lipocalin, β-defensin-2) expression, altered barrier (keratin 17, 79) functions, and a non-inflammatory Th17/IL-17 dominance in SGR skin compared to SGP. Regarding pro-inflammatory molecules (IL-1α, IL-6, IL-8, IL-33, TNF-α), similarly low levels were detected in both regions. Our data may explain the characteristic topographical localization of some immune-mediated and autoimmune skin disorders and we also propose that the term “healthy skin control sample,” widely used in experimental Dermatology, should only be accepted if researchers carefully specify the exact region of the healthy skin (along with the site of the diseased sample).

## Introduction

The skin exhibits several essential functions, including its role in formation and maintenance of the barrier. Unlike other epithelial surfaces, the skin has two major barrier elements, i.e., the stratum corneum and the tight junction layer. Besides these, it is also equipped with a complex network of cells and soluble mediators as part of the skin immune system (SIS) (1, 2). The anatomical and histological skin structure is characterized by major differences on distinct regions, due to the inhomogeneous thickness of the stratum corneum and, moreover, due to variable numbers of sebaceous, eccrine, and apocrine glands at different body sites, resulting diverse chemical milieu on the skin surface. Parallel to this varied chemical milieu, the skin microbiota has also been shown to exhibit remarkable differences on topographically distinct skin areas (3, 4); indeed, specific commensal flora have been associated with moist, dry, or sebaceous microenvironments.

On the other hand, the immunological barrier of the healthy skin is considered to be unified on the whole body surface—albeit recent indirect findings have challenged this dogma. In fact, the microbiota was shown to exert remarkable influence on the barrier’s immune function (5, 6).

Moreover, our previous study revealed that, similar to the aforementioned heterogeneity of the skin microbiota and chemical milieu, a fine topographical difference does exist in the expression pattern and activity of the SIS between sebaceous gland rich and sebaceous gland poor regions (SGR and SGP, respectively) of the human skin (7). Indeed, in SGR skin, higher, yet still homeostatic, non-inflammatory thymic stromal lymphopoietin (TSLP) expression was detected in epidermal keratinocytes (KC) which was regulated by linoleic acid, a major sebum component. Furthermore, significantly higher number of non-activated CD11c^+^ dendritic cells (DC), CD4^+^ T cells, and a characteristic cytokine expression pattern were detected in these areas. According to these results, we hypothesized that SGR skin might exhibit a distinct, non-inflammatory immune surveillance which is different from that found in SGP skin.

In the present study, as an extended continuation of our previous work, we aimed at in-depth analyzing the putative differences between SGR and SGP regions. For this, we performed comparative transcriptomic and subsequent pathway enrichment analyses, together with validation of selected molecules at the gene and protein levels. Here, we provide the first evidence that significantly enhanced innate [chemokine, antimicrobial peptide (AMP)] and distinct adaptive [dominant T helper (Th17) presence] immune responses and altered barrier [keratin (KRT) 17, 79] functions characterize SGR skin compared to SGP. Regarding pro-inflammatory cytokines [IL-1α, IL-6, IL-8, IL-33, tumor necrosis factor alpha (TNF-α)], similarly low levels could be detected in both healthy skin areas which implicates the lack of inflammation.

Beyond that our study provide new data on the characteristics of SIS and call the attention to the proper selection of healthy skin controls in research, several additional conclusions can also be deducted. The fact that region-specific differences exist in the composition of SIS allow to consider the pathogenesis of those inflammatory and autoimmune skin disorders that favorably localize to a given skin area (acne, rosacea, cutaneous lupus to SGR skin), from a new aspect. On the other hand, region-specific differences in skin barrier formation can underline the probable need to develop distinct barrier restoring strategies on distinct skin areas.

## Materials and Methods

### Skin Biopsies

Skin punch biopsies (0.5–1 cm^2^) were taken from normal skin of 20 healthy individuals (10 from SGP and 10 from SGR skin sites) undergoing plastic surgery after obtaining written, informed consent, according to the Declaration of Helsinki principles (Table 1). The study was approved by the local ethics committee of University of Debrecen, Hungary. All biopsies were cut into two pieces. For IHC, samples were paraffin-embedded, whereas for RT-PCR, samples were stored in RNAlater (Qiagen, Hilden, Germany) at −70°C until RNA isolation. After hematoxylin and eosin (H&E) staining, samples were sorted according to the number of sebaceous glands and were defined as SGP skin when containing *n* ≤ 1 sebaceous glands and as SGR skin when containing *n* ≥ 3 sebaceous glands in the field of view on 10× magnification in the microscope.

**Table 1:** Characteristics of the studied skin samples of healthy individuals.

Healthy individuals/patients	Sex	Age	Localization	Count of sebaceous glands
**SGP skin (*n* = 10)**				
SGP 1	M	77	Shin	−
SGP 2	M	85	Shin	−
SGP 3	F	72	Lower arm	−
SGP 4	F	81	Lower arm	−
SGP 5	M	40	Lower arm	−
SGP 6	F	72	Lower arm	−
SGP 7	F	86	Hand	−
SGP 8	F	56	Shin	−
SGP 9	M	64	Shin	−
SGP 10	F	56	Shin	−

MEAN AGE ± SD		68.9 ± 14.8		

**SGR skin (*n* = 10)**				
SGR 1	F	77	Heary scalp	+
SGR 2	M	62	Mandibula	++
SGR 3	F	57	Nose	+++
SGR 4	F	61	Nose	+++
SGR 5	F	42	Scapula	++
SGR 6	F	38	Chin	++
SGR 7	M	56	Shoulder	+++
SGR 8	M	47	Heary scalp	++
SGR 9	F	19	Face (central part)	+++
SGR 10	M	66	Face (lateral part)	+++

MEAN AGE ± SD		52.5 ± 16.5		

*Scoring of sebaceous gland count was performed according to the number and size of sebaceous glands in the field of view on 10× magnification: samples containing *n* ≤ 1 sebaceous gland were defined as negative (−), those containing *n* ≥ 3 sebaceous glands were defined as positive and scored in accordance with the area of sebaceous glands in percentage of dermal surface: (+) 5–15%; (++) 15–30%; and (+++) more than 30%*.

### RNA Isolation, Reverse Transcription, and Real-time Quantitative PCR

All samples were homogenized in Tri Reagent solution (Sigma-Aldrich, Dorset, UK) with Tissue Lyser (QIAGEN) using previously autoclaved metal beads (QIAGEN), and total RNA was isolated from the human skin tissues. The concentrations and purities of the RNA samples were measured by means of NanoDrop spectrophotometer (Thermo Scientific, Bioscience, Budapest, Hungary), and its quality was checked using Agilent 2100 Bioanalyser (Agilent Technologies, Santa Clara, CA, USA). In the reverse transcription step, 1ug of total RNA were reverse transcribed into complementary DNA (cDNA) using the high capacity cDNA Archive Kit (Invitrogen, Life Technologies, San Francisco, CA, USA) according to the manufacturer’s instructions and the indicated thermal protocol. Previously samples are treated with DNase I (Applied Biosystems, Foster City, CA, USA). QRT-PCR measurements was carried out in triplicate using pre-designed FAM-MGB assays as well as TaqMan^®^ Gene Expression Master Mix ordered from Applied Biosystems (Life Technologies). The following primers were used: PPIA (Hs99999904_m1), CCL2 (Hs00234140_m1), CCL3 (Hs00234142_m1), CCL19 (Hs00171149_m1), CCL20 (Hs00355476_m1), CCL23 (Hs00270756_m1), CCL24 (Hs00171082_m1), LCN2 (Hs01008571_m1), LOR (Hs01894962_s1), FLG (Hs00856927_g1), LCE1F (Hs00820275_sH), CLDN16 (Hs00198134_m1), KRT17 (Hs00356958_m1), and KRT79 (Hs00418343_m1). All reactions were performed with a LightCycler^®^ 480 System (Roche). Relative mRNA levels were calculated using either the comparative CT or standard curve methods normalized to the expression of PPIA mRNA.

### RNA Sequencing (RNASeq) Analysis

Complementary DNA library for RNASeq was generated from 1 µg total RNA using TruSeq RNA Sample Preparation Kit (Illumina, San Diego, CA, USA) according to the manufacturer’s protocol. Briefly, poly-A tailed RNAs were purified by oligodT-conjugated magnetic beads and fragmented on 94 C for 8 min, then 1st strand cDNA was transcribed using random primers and SuperScript II reverse transcriptase (Lifetechnologies, Carslbad, CA, USA). Following this step second strand cDNA synthesized, double stranded cDNA end repaired and 3′ ends adenylated then Illumina index adapters were ligated. After adapter ligation enrichment, PCR was performed to amplify adapter ligated cDNA fragments. Fragment size distribution and molarity of libraries were checked on Agilent BioAnalyzer DNA1000 chip (Agilent Technologies, Santa Clara, CA, USA). Concentrations of RNASeq libraries were set to 10 nM and 5 libraries were pooled together before sequencing. Single read 50 bp sequencing run was performed on Illumina HiScan SQ instrument (Illumina, San Diego, CA, USA) and 16–18 million reads per sample were obtained. CASAVA software was used for pass filtering and demultiplexing process. Sequenced reads were aligned to Human Genome v19 using TopHat and Cufflinks algorithms and bam files were generated. StrandNGS software was used for further statistical analysis. Bam files were imported and normalized using DESeq algorithm. To identify statistically significant gene expression patterns between conditions non-parametric Wilcoxon–Mann–Whitney test was used.

Library preparations, sequencing and data analysis were performed at the Genomic Medicine and Bioinformatics Core Facility of University of Debrecen.

RNAseq data have been deposited to Sequence Read Archive (SRA) database (https://www.ncbi.nlm.nih.gov/sra) under accession number SRP126212.

### Pathway Analyses

To map associated genes to their respective pathways, complex interactive pathway analysis was performed using the default analysis parameters of Ingenuity Pathway Analysis (IPA) software (Qiagen, Valencia, CA, USA) web-based application. Our input gene list contained the genes, which showed significantly different expression between SGR and SGP groups. The goal of the analysis was to predict overrepresented pathways, gene networks and upstream regulators (transcription factors, cytokines, chemokines), which help to characterize the functional and molecular differences between the two types of skin regions. The gene list was imported directly from StrandNGS software into the IPA to perform IPA Core Analysis with general settings: (1) fold change values were added as associated values to the analysis, (2) reference set: Ingenuity Knowledge Base (genes only), (3) gene symbols were used as identifiers, (4) species: *Homo sapiens*, (5) relationship to include: direct and indirect, (6) *p*-value cutoff: 0.05, (7.) includes endogenous chemicals, and (8) predict: (a) diseases and bio functions: (i) diseases and disorders, (ii) molecular and cellular functions, (iii) physiological system development and function, (b) canonical pathways, (c) molecules, and (d) upstream regulators.

A focused enrichment analysis was also performed on immune system-related genes revealed by IPA and those molecules which have been detected to be significantly differentially expressed by RT-PCR or immunohistochemistry (IHC) in our present and previous study (7) by ClueGo (v. 2.3.5) (8) and CluePedia (v. 1.3.5) (9) tool kits of Cytoscape (www.cytoscape.org) software (v. 3.5.1) (10) using gene ontology (GO) biological process (BP), GO immune system process (ISP), GO molecular function (MF), Kyoto encyclopedia of genes and genomes (KEGG), and reactome pathways databases. In our strict analysis, only significantly (*p* < 0,05) enriched pathways were visualized with an additional criterion that enriched terms should have contained at least nine genes from our input gene list. Regarding the statistical approach of the enrichment analysis by Cytoscape, a *p*-value of <0.05 and kappa coefficient of 0.4 were considered as threshold values and correction was performed by Benjamini–Hochberg test.

### IHC and Routine Staining

For IHC analyses, paraffin-embedded sections from patients and healthy controls were deparaffinized. Heat-induced antigen retrieval was performed and sections were pre-processed with H2O2 for 10 min. Sections were stained with antibodies against human S100A8 (rabbit polyclonal IgG [HPA024372]: Sigma-Aldrich), human lipocalin/NGAL [rabbit polyclonal IgG (PA5-32476): Invitrogen], human CCL2/MCP1 [mouse monoclonal IgG1 (NBP2-22115): Novus Biologicals, Littleton, CO, USA], human CCL20/MIP-3-α [mouse monoclonal IgG (LS-B7409): LifeSpan Biosciences, Seattle WA, USA], human KRT17 [rabbit polyclonal IgG (ab53707): Abcam], human loricrin [rabbit monoclonal IgG (NBP1-33610): Novus Biologicals], human FLG [mouse monoclonal IgG (ab17808): Abcam]. Subsequently, anti-mouse/rabbit (Dako) HRP-conjugated secondary antibody was employed. Before and after incubating with antibodies, washing of samples was performed for 5 min, 3 times in each step. Staining was detected with the Vector^®^ VIP and ImmPACT™ NovaRED™ Kit (VECTOR Laboratories, Burlingame, CA, USA). Sections were counterstained with methylene green. The detection of one protein was carried out on all sections in parallel at the same time to enable us to evaluate comparable protein levels. Positive, Ig, and isotype controls were also used to normalize staining against all proteins [mouse IgG (Covalab), rabbit immunoglobulin fraction (Dako)]. Skin specimens were also stained with H&E. Visual scoring of sebaceous glands’ count was performed by professional pathologist.

### Statistical Analysis

Statistical analyses were performed using GraphPad Prism software version 6 (GraphPad Software Inc., San Diego, CA, USA). Statistical comparisons of two groups were done using the unpaired *t*-test. Differences between the groups were demonstrated using mean ± SEM. *p* values <0.05 were considered statistically significant (*p < 0.05; **p < 0.01; ***p < 0.001).

## Results

### RNASeq and IPA Analyses Reveal Prominent Differences between SGR and SGP Skin Regions

#### RNA Sequencing

In order to explore the in-depth differences between SGR and SGP skin, RNASeq analysis was performed on whole skin lysates of 6 SGR and 7 SGP patients.

**Figure 1:**
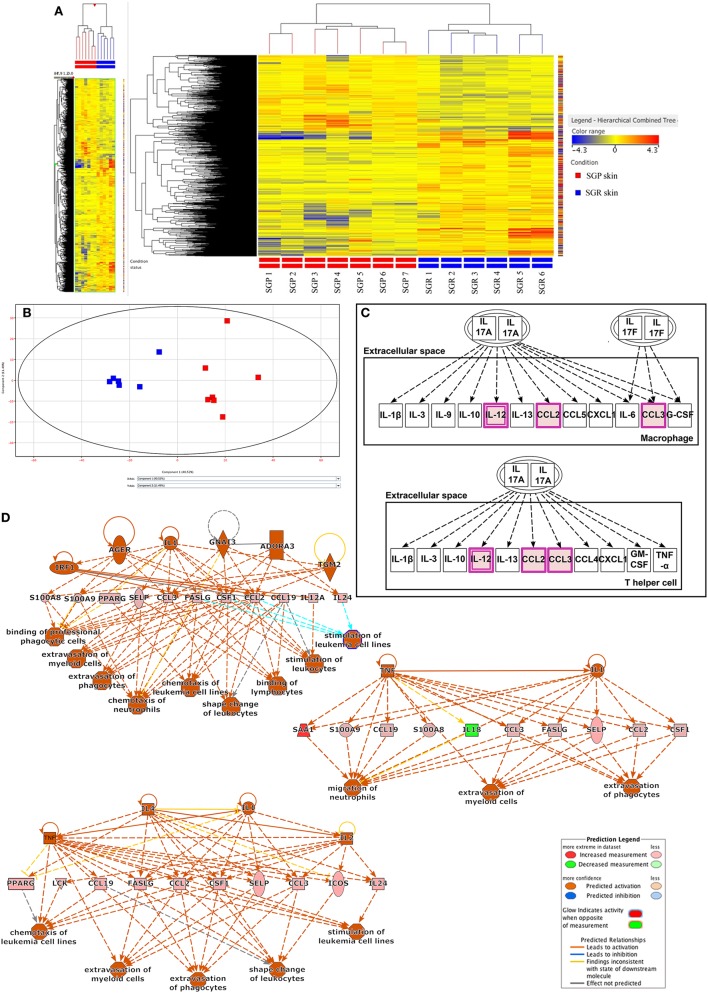
RNA Sequencing analyses revealed differences in innate and adaptive immune responses between sebaceous gland poor (SGP) and sebaceous gland rich (SGR) skin regions **(A)** Heat map was created by analyzing genes showing significantly different expression (*p* < 0.05) between SGR (*n* = 6) and SGP (*n* = 7) skin. By using StrandNGS software the two sample groups could be distinguished unambiguously based on the level of difference between the gene expression profiles of samples. On the heatmap, this can be seen based on the color scheme of the samples (red: higher expression, blue: lower expression). **(B)** Principal component analysis (PCA) of RNASeq data (also generated by StrandNGS) showing all samples. On PCA figure each dots represent one skin sample, the color of dots indicate sample type (red: SGP and blue: SGR) and the distance between the dots shows the level of difference between the gene expression profiles of samples. **(C)** On the basis of Ingenuity Pathway Analysis (IPA), when analyzing genes with significantly different expression between SGP and SGR skin, the first (i.e., the most significant) pathway exclusively related to the skin immune system (SIS) was the IL-17 related one. Genes marked with purple color were present in our gene list subjected to pathway analysis. **(D)** Regulatory IPA analysis identified three highly overlapping, SIS-related Regulator Effect Networks, in which both upstream regulators and downstream cellular responses were identified in relation to certain gene panels. These networks were linked to immune signaling processes and pathways, which also contained IL-17 related molecules, such as CCL2, S100A8, and S100A9.

StrandNGS software was applied to create the heatmap (Figure 1A) and the principal component analysis (PCA) figure (Figure 1B) of our RNASeq data. The heatmap, which was automatically generated by the software, aims to provide evidence on whether the two types of skin regions are distinguished based on the gene expression profiles of the samples derived from certain regions (SGP or SGR). Importantly, the heatmap clearly shows that the two regions are indeed unambiguously separated by the software. Similarly, the PCA figure (generated also by StrandNGS) also reflects the mentioned distinction between the two regions. In the PCA figure, each dot represents one individual skin sample, the color of the dots indicates the region (SGP or SGR), and the distance between the dots shows the level of difference between the gene expression profiles of the samples. On the basis of these, it is evident that dots with the same color created two distinct groups (red: SGP and blue: SGR) and that the distance between the differentially colored groups is prominent. Of further importance, the heatmap and the PCA figure also indicate that gene expression profiles of the samples belonging to the given (SGR or SGP) group were similar irrespective of the origin of the specimen in a certain skin region. In the heatmap, this can be seen based on the color scheme of the samples (red: higher expression, blue: lower expression), while in the PCA figure, dots representing one region can be found within relatively small distance compared to the dots from the other region.

Mann–Whitney non-parametric statistical test (*p* < 0.05) was then performed to determine differential gene expression profiles of SGR and SGP samples. With this analysis, 1,082 genes were found to be differentially (and significantly) expressed in SGR compared with SGP skin; out of these, 672 genes showed higher, whereas 411 genes exhibited lower expressions in the SGR tissues (Table S1 in Supplementary Material).

#### Ingenuity Pathway Analysis

Using the IPA software, the above 1,082 genes were then subjected to two different types of functional, standard, non-restricted pathway analyses: canonical pathway analysis and regulatory analysis.

First, we performed a non-restricted canonical pathway analysis, which revealed 40 significantly enriched terms (canonical pathway were automatically arranged by IPA based on the level of significance; see Figure S1 in Supplementary Material.). Of these 40 canonical pathways, the first 14 in the significance ranking list were all related to lipid metabolism (such as LXR/RXR Activation, FXR/RXR Activation, Stearate Biosynthesis I, and so on). This was not surprising at all since the *per definition* anatomical differences (presence or lack of sebaceous glands) of the two skin regions predisposed these results. The first (i.e., the most significant) pathway exclusively related to the SIS was the IL-17 related one (Figure 1C). Besides IL-17 signaling, the only pathway which could be partially connected to skin immune functions was “LPS/IL-1 mediated inhibition of RXR function.”

Next, regulatory IPA analysis was applied; it revealed eight signaling networks in which both upstream regulators and downstream cellular responses were identified in relation to certain gene panels. Three of these networks were linked to immune signaling processes and pathways, which also contained IL-17 related molecules, such as CCL2, S100A8, and S100A9 (Figure 1D) whereas all the other five pathways were somehow related to lipid metabolism (Figures S2A–E in Supplementary Material).

### Further Analysis and Validation Strategies

Since, both the two pathway analyses and our previous results highlighted that differences do exist in the expression of innate and adaptive immune and also permeability barrier molecules between SGP and SGR region. These results encouraged us to select genes for further RT-PCR validation on an extended number of samples (SGP: *n* = 10, SGR: *n* = 10) far beyond our RNASeq data. Namely, based on literature data regarding the most important AMP, chemokine, cytokine, permeability barrier, and adaptive immune molecules of SIS five groups of genes were formed: (1) AMPs, (2) Chemokines, (3) Barrier genes, (4) Pro-inflammatory molecules (Table 2), and (5) T helper-related molecules (Table 3).

**Table 2:** Expressions of innate immune molecules, as assessed by RNASeq and quantitative real-time PCR (qRT-PCR), including antimicrobial peptides (AMPs), chemokines, pro-inflammatory molecules and barrier genes in SGR and SGP skin samples.

AMPs	qRT-PCR		RNA Seq	
	*p*	SGR/SGP (FC)	*p*	SGR/SGP (FC)
S100A7	**0.002**	40.36	0.076	3.14
S100A8	**0.028**	14.99	**0.017**	4.28
S100A9	**0.021**	17.49	**0.024**	3.50
DEFB4B (hBD-2)	**0.0002**	UDL in SGP	0.154	2.26
LCN2	**0.0003**	12.12	0.074	4.02
CAMP	0.329	1.83	0.384	1.41

**Chemokines**	**qRT-PCR**		**RNA Seq**	
	***p***	**SGR/SGP (FC)**	***p***	**SGR/SGP (FC)**

CCL2	**0.032**	1.78	**0.0001**	2.13
CCL3	**0.037**	3.58	**0.030**	2.64
CCL19	**0.005**	3.31	**0.026**	2.96
CCL20	**0.047**	3.44	0.116	2.77
CCL23	**0.013**	4.34	**0.044**	3.24
CCL24	**0.038**	3.68	0.073	2.32

**Pro-inflammatory molecules**	**qRT-PCR**		**RNA Seq**	
	***p***	**SGR/SGP (FC)**	***p***	**SGR/SGP (FC)**

TLR2	0.150	3.35	0.209	1.36
TLR3	0.468	1.17	**0.027**	−1.74
TLR4	0.267	2.29	0.929	−1.04
NLRP3	0.291	1.18	0.389	1.54
IL-1A	0.127	2.35	0.515	1.49
IL-1B	**0.003**	3.10	0.288	1.80
IL-6	0.120	3.10	0.192	−1.77
IL-8	0.414	2.87	0.429	−1.14
IL-33	0.110	1.36	0.720	1.24
TNFA	0.267	1.87	0.147	2.02

**Barrier genes**	**qRT-PCR**		**RNA Seq**	
	***p***	**SGR/SGP (FC)**	***p***	**SGR/SGP (FC)**

LOR	0.434	−5.53	0.101	−2.30
FLG	0.092	−3.98	0.409	−1.40
LCE1F	0.168	−1.29	0.051	−2.27
CLDN1	0.122	−1.46	0.054	−2.02
KRT17	**0.002**	4.36	0.103	2.59
KRT79	**0.027**	2.29	**0.005**	7.72

*Fold change (FC) values are calculated by dividing the expression levels measured in SGR by those of SGP (SGR/SGP). The *p* values of significantly differentially expressed genes were highlighted in bold*.

*CAMP, cathelicidin; CCL, chemokine (C-C motif) ligand; CLDN, claudin; DEFB, defensin beta; FLG, filaggrin; IL, interleukin; KRT, keratin; LCE, late cornified envelope; LCN, lipocalin; LOR, loricrin; NLR, nod-like receptor; S100, S100 calcium-binding protein; SGP, sebaceous gland poor; SGR, sebaceous gland rich; TLR, toll-like receptor; TNF, tumor necrosis factor; UDL, under detection limit*.

**Table 3:** Expressions of T helper-related molecules, as assessed by RNASeq and Quantitative real-time PCR (qRT-PCR) in SGR and SGP skin samples.

Th1 markers	qRT-PCR		RNA Seq	
	*p*	SGR/SGP (FC)	*p*	SGR/SGP (FC)
IL-12B		UDL	1.000	1
TBX21*	0.434	1.11	0.124	1.81
IFNG*		UDL	0.937	−1.02
TNFA	0.267	1.87	0.147	2.02

**Th2 markers**	**qRT-PCR**		**RNA Seq**	
	***p***	**SGR/SGP (FC)**	***p***	**SGR/SGP (FC)**

IL-13*		UDL	0.272	−1.45
GATA3*	0.327	1.10	0.399	−1.44

**Th22 markers**	**qRT-PCR**		**RNA Seq**	
	***p***	**SGR/SGP (FC)**	***p***	**SGR/SGP (FC)**

AHR	**0.006**	3.48	0.738	1.16
IL-22		UDL	0.292	1.13

**Th17 markers**	**qRT-PCR**		**RNA Seq**	
	***p***	**SGR/SGP (FC)**	***p***	**SGR/SGP (FC)**

IL-1B	**0.003**	3.10	0.288	1.80
IL-6	0.120	3.10	0.192	−1.77
IL-23A	**0.002**	UDL in SGP	0.979	1.01
TGFB1	0.432	−1.21	1.000	1
RORC*	0.300	1.37	0.450	−1.15
IL-10*	0.079	3.26	0.336	1.32
IL-17A*	**0.0003**	UDL in SGP	1.000	1
CCL20	**0.047**	3.44	0.116	2.77

*Fold change (FC) values are calculated by dividing the expression levels measured in SGR by those of SGP (SGR/SGP). The *p* values of significantly differentially expressed genes were highlighted in bold. qRT-PCR expression values of molecules marked with asterisk have been published in our previous study (7)*.

*AHR, aryl hydrocarbon receptor; CCL, chemokine (C-C motif) ligand; GATA, GATA-binding protein; IFN, interferon; IL, interleukin; ROR, RAR-related orphan receptor; SGP, sebaceous gland poor; SGR, sebaceous gland rich; TBX, T-box; TGF, transforming growth factor; TNF, tumor necrosis factor; UDL, under detection limit*.

Since we were interested in defining whether the results (tendency and level of changes in the expression of selected genes between the two regions) of the two mRNA based methods (RNASeq and RT-PCR) were similar, mRNA expression levels of genes detected by RT-PCR were compared to that of our previous RNASeq data set (Tables 2 and 3). The comparison revealed that expressions of nearly all of the investigated genes altered in the same direction detected by the two distinct methods. Moreover, in several cases, on the extended number of samples significant differences could be detected by using RT-PCR in spite of the fact that expression levels of these certain genes (S100A7, DEFB4B, LCN2, CCL20, CCL24, IL-1B, and KRT17) did not differ significantly in the RNASeq data set. Notably, the complementary nature of the two distinct methods has already been documented by presenting, e.g., that RT-PCR was able to detect significant gene expression differences where RNASeq indicated only tendencies (11).

Finally, to verify the differential expressions in the two skin regions also at the protein level, certain molecules were subjected to IHC and image analyses.

### Prominent Differences in Innate Immune Responses between SGR and SGP Skin

#### Expressions of AMPs Are Significantly Higher in SGR Skin

**Figure 2:**
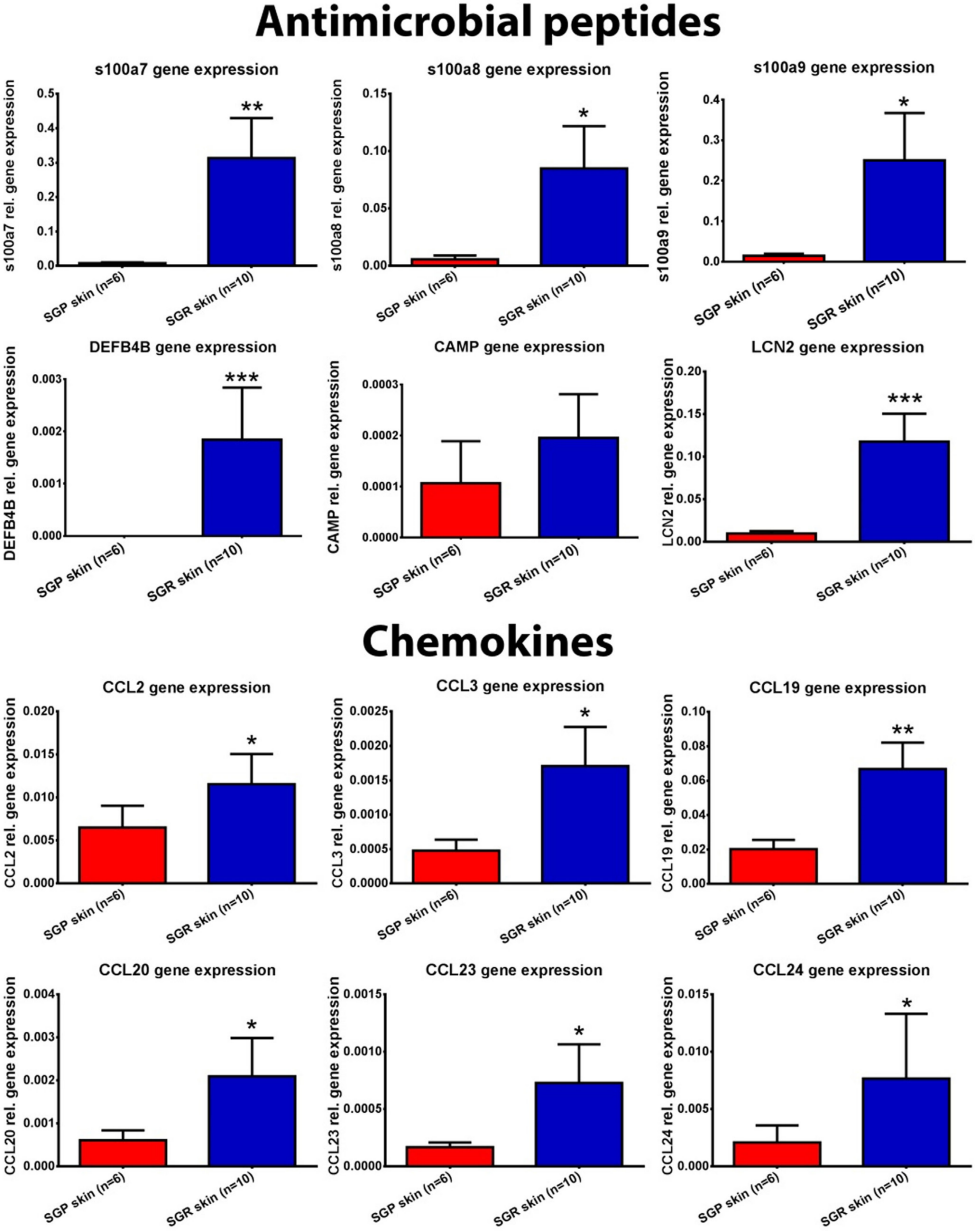

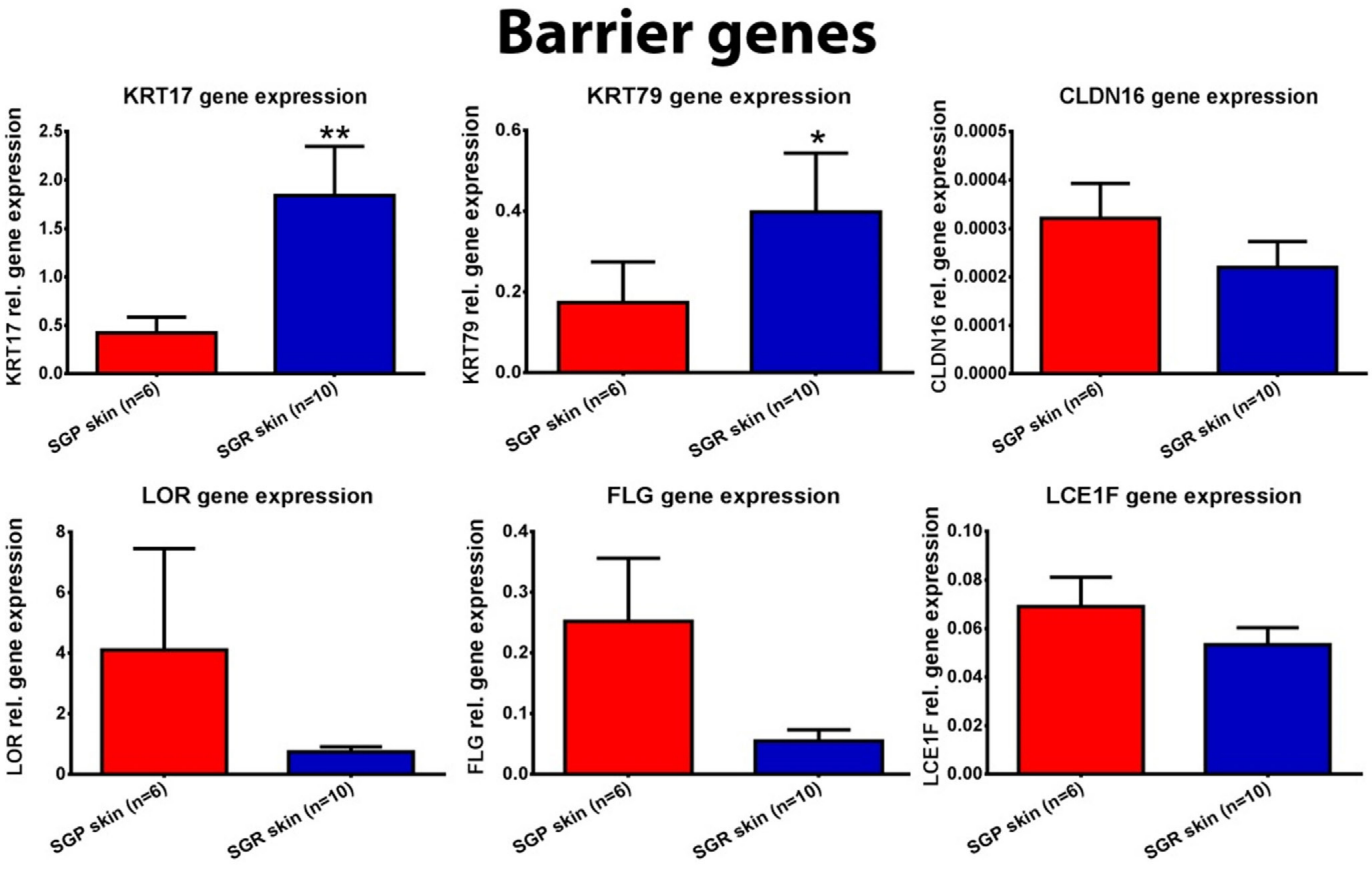
Expression of antimicrobial peptides (AMP), chemokines, and barrier genes in sebaceous gland poor (SGP) and sebaceous gland rich (SGR) regions examined by quantitative real-time PCR (qRT-PCR). The graphs show the mean ± SEM of measured mRNA transcript levels (*p < 0.05; **p < 0.01; ***p < 0.001. as determined by Mann–Whitney *U*-test). Abbreviations: CAMP, cathelicidin; CCL, chemokine (C-C motif) ligand; CLDN, claudin; DEFB, defensin beta; FLG, filaggrin; KRT, keratin; LCE, late cornified envelope; LCN, lipocalin; LOR, loricrin; S100, S100 calcium-binding protein; SGP, sebaceous gland poor; SGR, sebaceous gland rich.

**Figure 3:**
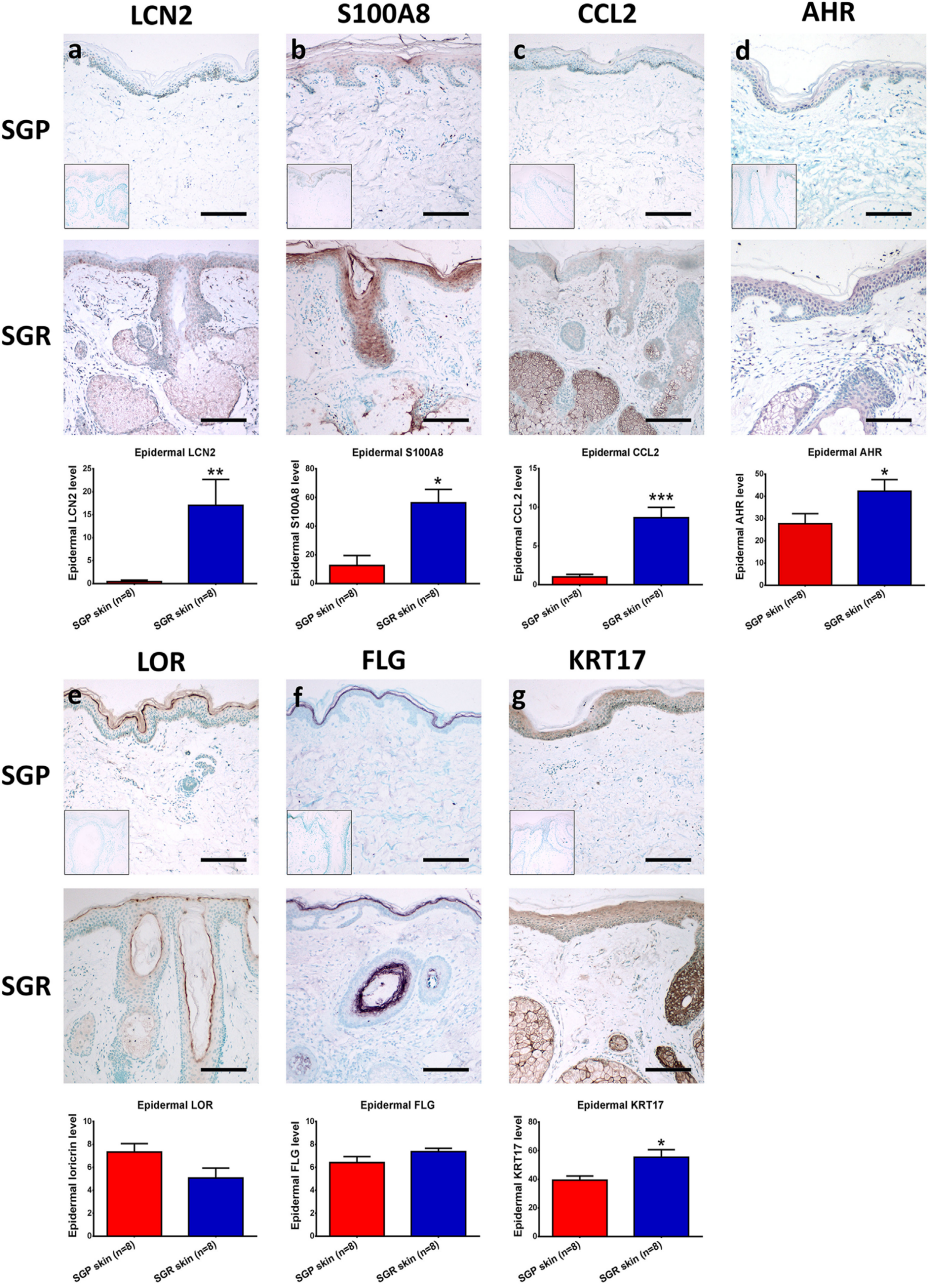
Prominent differences in the expressions of innate immune and barrier molecules between sebaceous gland poor (SGR) and sebaceous gland rich (SGP) skin regions. Representative images for immunostaining and quantification of epidermal levels of **(A)** LCN2, **(B)** S100A8, **(C)** CCL2, **(D)** AHR, **(E)** LOR, **(F)** FLG, and **(G)** KRT17 in SGP and SGR skin sections. Images of negative control stainings are shown in the bottom left corner of SGP immunostainings. Size bars = 100 µm. The graphs show the mean ± SEM of measured protein levels (*p < 0.05; **p < 0.01; ***p < 0.001, as determined by Mann–Whitney *U*-test). Abbreviations: AHR, aryl hydrocarbon receptor; CCL, chemokine (C-C motif) ligand; FLG, filaggrin; KRT, keratin; LCN, lipocalin; LOR, loricrin; S100, S100 calcium-binding protein; SGP, sebaceous gland poor; SGR, sebaceous gland rich.

First, we aimed to assess the expressions of AMPs since, besides their antibacterial actions, their role as alarmin molecules could also emerge in healthy skin regions. By employing qRT-PCR, gene expression levels of S100A7 (psoriasin), S100A8, S100A9, human β-defensin-2 [hBD-2 (DEFB4B)] and lipocalin (LCN2) were high and significantly increased in SGR skin, whereas these molecules only weakly expressed in SGP skin. Using RNASeq, expressions of all AMPs were elevated in SGR skin; the increase regarding S100A8 and S100A9 was found to be significant. Expression of cathelicidin (CAMP) was very low both in SGP and SGR samples with a slight tendency of increase in SGR skin (Table 2; Figure 2). In the cases of S100A8 and LCN2, immunostaining was also performed and revealed significantly higher protein levels in SGR samples for both AMPs (Figure 3). LCN2 could not be detected in SGP samples; in SGR skin, the apical layer of the epidermis and sebocytes showed slight positivity and its strongest expression was found in follicular KCs. Immunostaining of S100A8 also revealed prominent differences. This protein could be detected at low levels in SGP skin; however, it was present at high levels in the upper layers of epidermal KCs, in follicular KCs and in sebocytes of SGR skin.

#### Expressions of Chemokines Are Significantly Higher in SGR Skin

Then, the assessment of certain chemokines (CCL2, CCL3, CCL19, CCL20, CCL23, and CCL24), produced by innate immune cells (KCs, DCs, macrophages), was performed. In SGP skin, by using qRT-PCR, expressions of CCL2, CCL19, and CCL20 were well detectable but levels of CCL3, CCL23, and CCL24 were very low. Of great importance, qRT-PCR revealed significantly higher levels of all investigated chemokines in SGR skin (Table 2; Figure 2). Likewise, prominently—and in the cases of CCL2, CCL3, CCL19, and CCL23, significantly—higher expressions of these molecules in SGR skin were also verified by RNASeq. We also investigated the expressions of CCL2 and CCL20 at the protein level by IHC. CCL2 was highly expressed in the sebaceous glands in SGR skin. Although the epidermal CCL2 positivity was weak in both skin types, image analysis revealed significantly higher expression in the SGR region (Figure 3). By IHC, CCL20 could not be detected either in KCs or in sebocytes in both skin regions (data not shown).

#### No Significantly Different Expression Patterns between SGR and SGP Skin Are Detected Regarding Innate Immune System Receptors and Pro-inflammatory Cytokines

Since marked regional differences of skin microbiota have been described previously (3, 4, 12), we also assessed the expressions of well-known molecular sensors of KCs, namely toll-like receptor (TLR) 2, TLR3, TLR4, and nod-like receptor 3 (NLRP3). RNASeq revealed similar gene expression levels of the investigated receptors in SGR and SGP skin except TLR3, which showed a significant increase in SGR. qRT-PCR measurements confirmed the rather insignificant differences in expressions of the above receptors (Table 2).

Next, specific mRNA transcript levels of pro-inflammatory cytokines [namely IL-1α, IL-1β, IL-6, IL-8, IL-33, and tumor necrosis factor alpha (TNF-α)] were compared. RNASeq data showed similar expressions in the two regions. Likewise, qRT-PCR revealed no significant differences between their mRNA levels, except for the significantly higher expression of IL-1β in SGR samples (Table 2).

### Altered Barrier Gene Expression in SGR Compared to SGP

We were also interested in uncovering the potential differences in expressions of key molecules involved in the formation and maintenance of the epidermal barrier. By qRT-PCR, expressions of loricrin (LOR), late cornified envelope 1 F (LCE1F), claudin 16 (CLDN16), and filaggrin (FLG) showed tendencies of decreased expression in the SGR skin, whilst KRT17 and KRT79 expressed at higher levels in the SGR samples (the latter ones were found to be significantly higher). The directions of differential expressions were confirmed by RNASeq and, in the cases of KRT79, the difference was significant (Table 2; Figure 2). KRT17, LOR, and FLG were also investigated at the protein level by IHC. KRT17 was present in significantly higher levels in SGR skin compared to SGP (Figure 3). In SGP skin, it was present in the upper layers of the epidermis; by contrast, in SGR samples, its expression was detected in the whole epidermis with the highest expression in the upper layers. Interestingly, the strongest immunoreactivity was found in follicular KCs, although sebocytes also showed notable positivity in SGR skin. Regarding FLG and LOR, no significant differences were identified between SGP and SGR regions, although the expression of LOR showed a tendency of decrease in SGR skin. Both proteins could be detected continuously with strong positivity in the granular and subcorneal layers of the epidermis. In SGR skin, hair follicle KCs also showed FLG- and LOR-positivity (Figure 3).

### SGR Skin Is Characterized by a Th17/IL-17 Pathway Dominance

#### Expressions of Th1, Th2, and Th22 Molecules Are Negligible and Similar in the Two Skin Regions

As a next step, we compared different T-cell subsets in SGR and SGP skin samples by investigating the expression of their signature and maturation cytokines, as well as their transcription factors. Gene expressions of molecules characteristic to Th1 (IL-12B, TBX21, IFN-γ, TNF-α), Th2 (IL-13, GATA3), and Th22 [aryl hydrocarbon receptor (AHR), IL-22] cells were not different in the two skin regions. By qRT-PCR analysis, IL-12B and IL-22 were undetectable in either area, whereas expression of AHR was significantly higher in SGR skin. The qRT-PCR investigation of TBX21, IFN-γ, IL-13, and GATA3 was already performed in our previous study (7), and was not re-evaluated in the current work (indicated in Table 3). To reveal what could be responsible for the differential expression of AHR between the two skin regions, IHC was also performed. Immunostaining of AHR also showed significantly higher protein levels in SGR samples; AHR was mainly expressed by KCs in their nucleus, but cytoplasmic staining in the epidermis was also detectable. A few cells in the dermis were also found positive for AHR (Figure 3).

#### Th17-Related Genes Exhibit Higher Expression in SGR Skin

Although RNASeq data alone did not reveal significant differences in the expression of Th17-related genes (IL-1β, IL-6, RORC, IL-23A, IL-17A, CCL20, IL-10, and transforming growth factor beta), as shown above, the in-depth bioinformatics pathway analyses have identified the Th17 pathway as a significantly enriched term (Figure 1C). Therefore, we further assessed the expression of these Th17-molecules by qRT-PCR. Importantly, in perfect agreement with our previous findings (7) which revealed significantly higher expression of IL-17A (but not of RORC and IL-10) in SGR skin, markedly and significantly (*p* < 0.05) elevated levels of IL-1β, IL-23A, and CCL20 as well as tendency of higher expression for IL-6 were detected in SGR compared to SGP skin (Table 3).

### Focused Pathway Analysis Revealed the Central Role of IL-17 Pathway in SGR Skin

As a final step, we performed another in-depth bioinformatics pathway analysis with those immune system-related molecules which had been shown significantly different expression either at the gene level (by RNA Seq or RT-qPCR) or at the protein level (by IHC) in our present and previous studies (i.e., AHR, CCL2, CCL3, CCL19, CCL20, CCL23, CCL24, CCR8, CD48, CD5, CSF1, DEFB4B, FASLG, ICOS, IFITM1, IFRD2, IL1B, IL10, IL10RA, IL12A, IL17A, IL18, IL23A, IL24, KRT17, KRT79, LCN2, PPARG, S100A7, S100A8, S100A9, SAA1, SAA2, SAA2-SAA4, SELP, TLR3, TSLP). The analysis was performed by ClueGo application of the Cytoscape software using GO BP, GO ISP, GO MF, KEGG, and Reactome Pathways databases.

**Figure 4:**
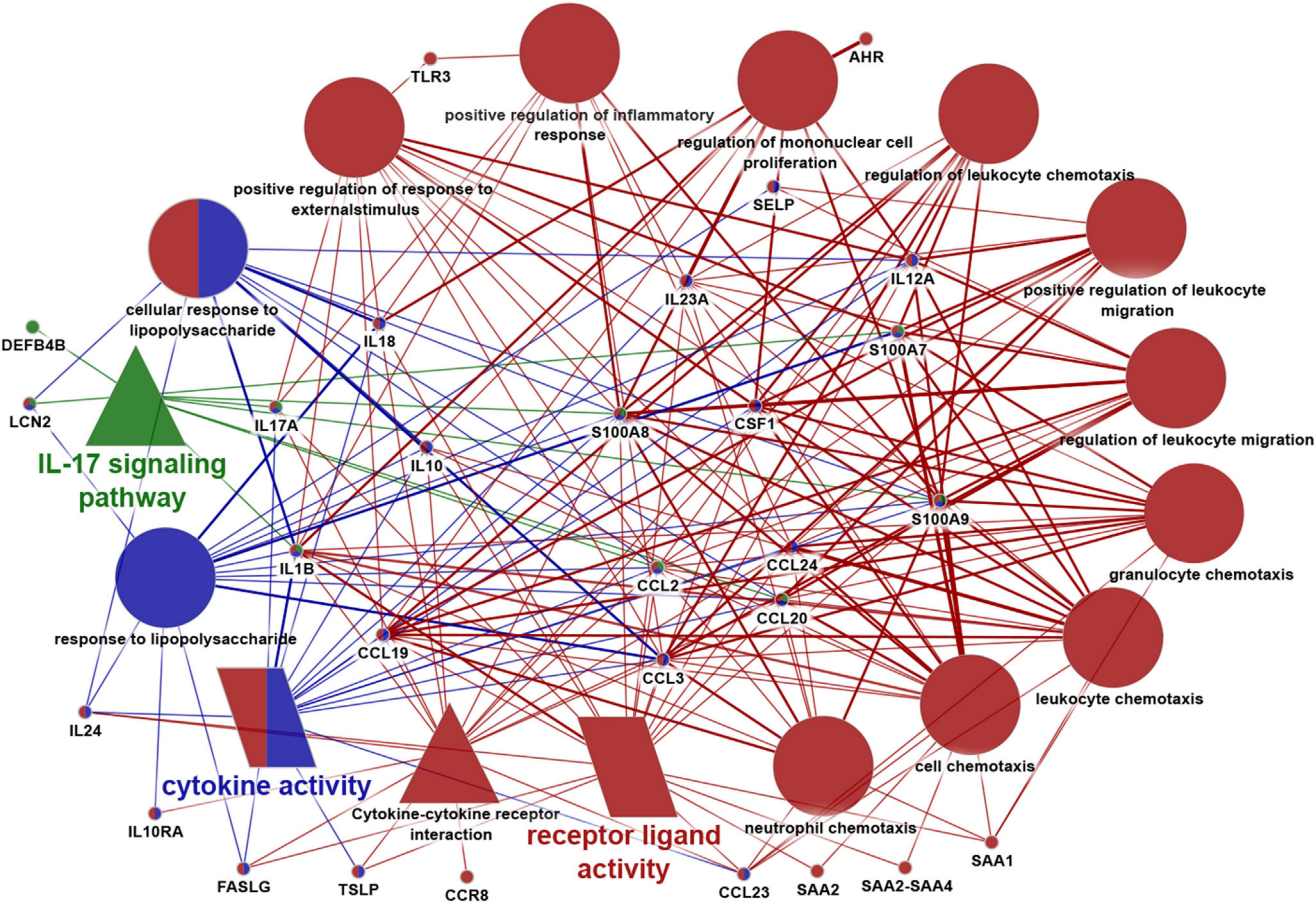
Data visualization of focused enrichment analysis. Immune system-related genes revealed by Ingenuity Pathway Analysis and those molecules that have been detected to be significantly differentially expressed by RT-PCR or immunohistochemistry in our present and previous study were all subjected to a new focused enrichment analysis performed by ClueGo application of Cytoscape software. Only significantly (*p* < 0,05) enriched pathways are shown. Additional criteria of the analysis: enriched terms should have contained at least nine genes from our input gene list. Symbols of terms represent different databases [circle: gene ontology (GO) Biological Process, triangle: Kyoto encyclopedia of genes and genomes, and parallelogram: GO molecular function]. Different colors represent the relationship between the terms based on the similarity of their associated genes (one color represents one group). Terms marked with more colors belong to more groups. Similarly, genes marked with more colors belong to more terms. The degree of connectivity between terms (edges) and genes is presented by the thickness of lines.

Importantly, by using ClueGo, the result of IPA analysis could be confirmed; indeed, IL-17 signaling pathway was found to be one of the most significantly enriched terms. Besides this pathway, multiple genes exhibiting roles in cytokine activity, cytokine–cytokine receptor activity, positive regulation of response to external stimulus, leukocyte chemotaxis, etc. were also identified among the significantly enriched pathways (Figure 4). These data, therefore, further confirmed that characteristic differences in activities/levels of the SIS could be defined between SGR and SGP skin region of the human body.

## Discussion

In this study, we performed extended and comparative analyses of innate and adaptive immune and also of barrier functions of SGR and SGP healthy skin, since earlier data challenged the unified nature of SIS. During the evaluation, significantly differentially expressed genes between SGR and SGP samples turned up in relatively high numbers, considering that two healthy skin regions were investigated. Furthermore, IPA canonical pathway analysis highlighted the importance of IL-17 signaling in the SGR region. These findings empowered/encouraged us to study in detail the IL-17-influenced innate immune and barrier milieu in the mentioned two healthy skin regions. Indeed, we employed complementary techniques and analyzed five molecular group characteristics to and markers of various skin functions. Albeit the reported data were mostly collected during transcriptomics analyses and, to lesser extent, from immunolabeling (hence future, detailed proteomics and functional studies are demanded and warranted), we found that there are indeed marked immunotopographical and barrier differences between the SGP and SGR regions of the human skin.

Assessment of expressions of AMPs revealed remarkable differences between SGR and SGP skin. AMPs are prominent effector mediators of the innate immune system, which have far more functions than their antimicrobial activity as they play regulatory roles in angiogenesis, wound healing, cell proliferation, and differentiation; moreover, they exert immune-modulatory actions such as stimulation of cytokine and chemokine production (13–16). Previous studies showed that hBD-1, hBD-2, hBD-3 and human CAMP were detectable at low levels in the differentiated epidermal layers of healthy skin (17, 18), while RNase7 was highly expressed by healthy KCs found by IHC (19) and S100A7 was considered to be one of the principal AMPs in normal skin (20). It must be noted that in these previous investigations, the origin of healthy skin samples has not been specified (21–26). Only one workgroup examined the regional presence of some AMPs (S100A7, hBD-3, and RNase7) in distinct healthy skin area. They found that all these proteins were expressed in higher amounts in the forehead (characteristic SGR regions) compared to lower leg (characteristic SGP region) shown by IHC (27), which data are in perfect agreement with our current results. Besides S100A7, hBD-2 and CAMP, S100A8, S100A9, and LCN2 were found to be undetectable by both RT-PCR and IHC or were not investigated previously in healthy skin (23, 25). In our present study, low levels of these AMPs (S100A7, A8, A9, CAMP, hBD-2, LCN2) were detected in SGP skin. Importantly, expressions of all of them, except CAMP, were found to be significantly higher in SGR skin. Moreover, S100A8 and LCN2 could be identified by IHC in both regions, with a significantly higher level in SGR skin.

Our knowledge about the investigated chemokines, mainly derived from innate immune cells (28–31) in healthy skin is quite incomplete. According to our data, in SGP samples, CCL3, CCL23, and CCL24 were hardly measurable, whereas CCL2, CCL19, and CCL20 expressions were detected in higher levels. Importantly, all six investigated chemokines were highly expressed in SGR samples, and their expressions were significantly higher compared to SGP samples. Previously, Nakayama et al. described a low expression of CCL20 in healthy skin by immunostaining without indicating the investigated region, whereas Nagao et al. were unable to visualize either CCL2 or CCL20 by immune fluorescent staining (32, 33). We found that albeit sebaceous glands showed a prominent staining of CCL2 resulting significantly higher CCL2 protein expression in SGR skin, the immunoreactivity of both CCL2 and CCL20 was very low or absent in the epidermis. As these chemokines mainly target T cells and also affect DCs and macrophages (26, 28–30), these data correlate well with our previous observation that T cells and DCs are present at significantly higher numbers in SGR skin (7).

In the case of the most important KC sensors (TLR2, TLR3, TLR4, NLRP3) and pro-inflammatory cytokines produced mainly by innate immune cells (IL-1α, IL-6, IL-8, IL-33, TNF-α), no significant differences were found between SGR and SGP skin; these findings were not surprising at all since healthy skin samples were compared. The only exception was the significantly higher level of IL-1β in SGR skin, which probably plays a role in establishing the later discussed Th17/IL-17 cytokine milieu of this region (34, 35).

Expressions of the late-terminal epidermal differentiation markers (LOR, LCE1F, FLG) as well as of the tight junction molecule CLDN16 were mostly lower in SGR than in SGP skin (albeit the difference was statistically insignificant); this may suggest that the epidermal barrier could be somewhat weaker in the SGR regions. Actually, this is supported by previous reports showing that the degree of transepidermal water loss, the increase of which correlates well with impaired barrier functions (36), is higher in characteristic SGR regions (different facial sites) vs. characteristic SGP regions (forearm, arm) (37, 38). By contrast, mRNA levels of KRT17 and KRT79 were significantly higher in the SGR skin; moreover, KRT17, which is usually expressed in basal cells of epithelia (such as in SGP skin), is markedly overexpressed in all layers of the epidermis in the SGR regions. Of further importance, previous studies have found that cytokines related to Th17/Th22 pathways (IL-17, IL-22) were shown to upregulate the epidermal expression of KRT17 detected by immunofluorescent staining and RT-PCR (39–41) and downregulated the level of LOR at the mRNA level detected by gene array (42). Therefore, these alterations in barrier molecules of KCs may be the result of the later discussed Th17 cell/IL-17 cytokine milieu of SGR skin regions.

Of greatest importance, however, expressions of components of Th17 signaling [(Th17 maturation cytokines and a Th17 effector chemokine) (43)] were markedly and, in multiple cases significantly (IL-1β, IL-23A, CCL20) higher in SGR skin compared to SGP. These results correlated well with our previous data, when we were able to detect significantly higher mRNA and protein expression of IL-17A found by RT-PCR and IHC (7), thus in this study we could reconfirm the presence of Th17 cells in SGR skin. Notably, not just the presence, but also the influence of IL-17 in SGR skin was observed, since the above detailed differences in the expression of AMPs, chemokines and barrier molecules between SGR and SGP can be explained well by the effect of IL-17. It was previously shown that IL-17 can upregulate the cutaneous expressions of IL-1β, hBD-2, CAMP, S100A7, S100A8, S100A9, LCN2, CCL2, CCL20 (24, 42), and KRT17 (39, 41) at the mRNA level detected by microarray and RT-PCR as well, whereas it can downregulate LOR in KCs (42). Moreover, our focused pathway analysis, intended to categorize significantly differentially expressed, immune system-related molecules into functional groups, could also confirm the important role of these molecules in the maintenance of SGR skin region-specific immune milieu. However, it should be firmly emphasized that the detected effect of IL-17 in SGR skin appears to be homeostatic and not inflammatory, since the expression of neutrophil chemoattractants (such as CXCL1, 3, 5, 6, and 8, as determined by RNASeq analysis in this study), the production of multiple pro-inflammatory molecules (see Table 2), and the degree of neutrophil infiltration [see our previous study (7)], was not significant in SGR skin. Interestingly, among the pro-inflammatory molecules, IL-1β was the only one which exhibited higher expression in SGR; we think that the higher IL-1β level can promote Th17 cell development and can contribute to IL-17 milieu, as detailed previously (44).

**Figure 5:**
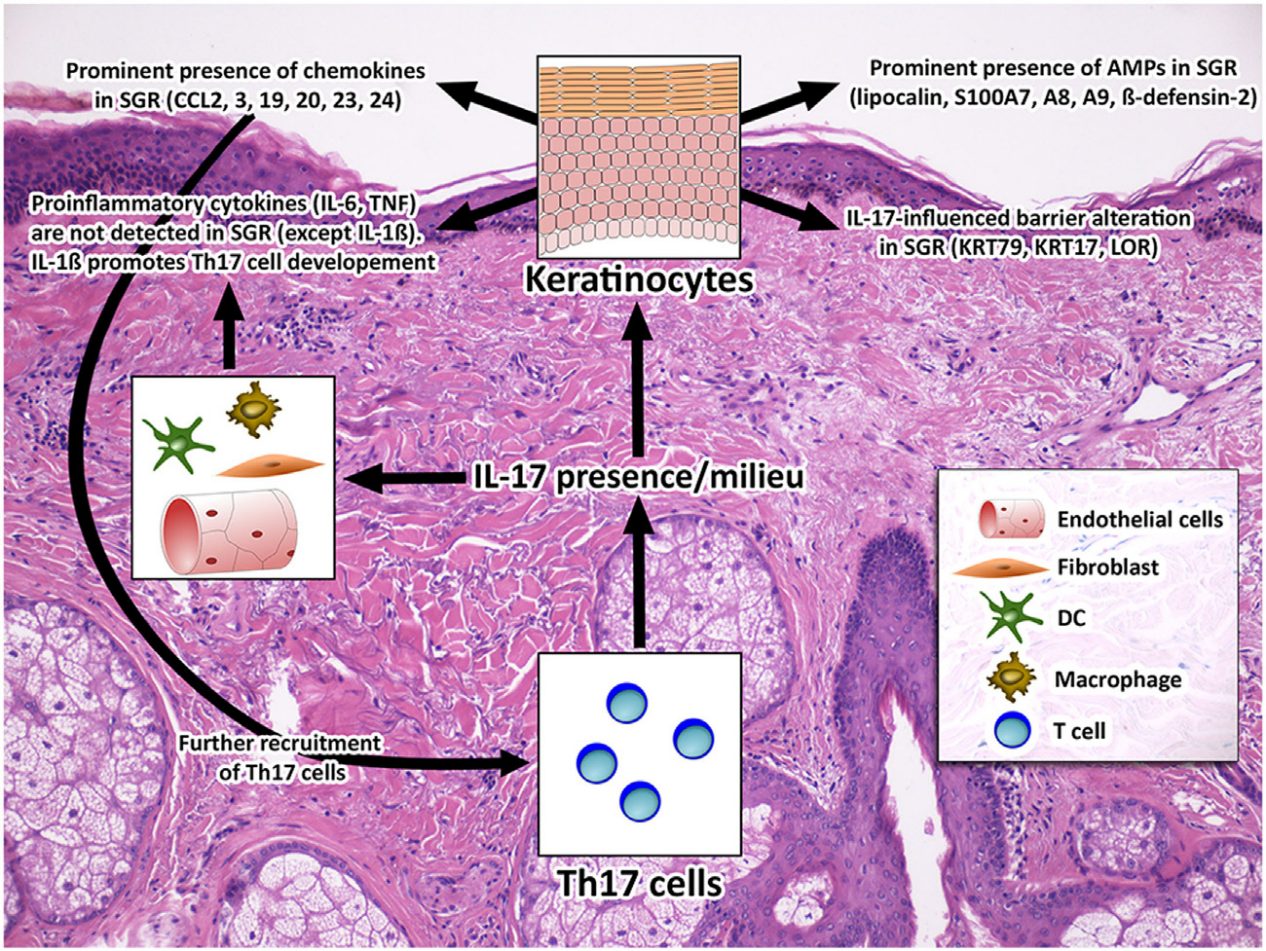
Healthy SGR skin is characterized by a non-inflammatory T helper 17 (Th17)/IL-17 milieu. The non-inflammatory Th17/IL-17 milieu of SGR skin, which was detected in this study, is combined with significantly altered expression of AMPs (S100A7, A8, A9, hBD-2, and lipocalin), chemokines (CCL2, 3, 19, 20, 23, and 24) and barrier molecules (KRT17, KRT79, LOR). From the above molecules S100A7, A8, A9, hBD-2, lipocalin, CCL2, 20, KRT17, and LOR have been proven to be IL-17-related (24, 39, 41, 42, 50). By contrast, the inflammatory IL-17 effects, such as the expression of neutrophil chemoattractants (RNA Sequencing analysis did not reveal significant differences of CXCL1, 3, 5, 6, 8 expression), neutrophil infiltration [see in our previous study (7)], and the production of pro-inflammatory cytokines (IL-6, TNF) are not characteristic to this region, suggesting that IL-17 has a distinct, homeostatic role in healthy SGR skin. Among the pro-inflammatory molecules, IL-1β (44) was the only one with an increased expression in SGR which may promote Th17 cell development and can contribute to IL-17 milieu in SGR skin. Abbreviations: AMP, antimicrobial peptide; CCL, chemokine (C-C motif) ligand; CXCL, chemokine (CXC motif) ligand; DC, dendritic cell. IL, interleukin; KRT, keratin; LOR, loricrin; S100, S100 calcium-binding protein, SGR, sebaceous gland rich; TNF, tumor necrosis factor.

Along these lines, we propose that the notable differences in skin immune and barrier parameters between SGR and SGP regions are connected to the distinctions in the composition of microbiota and skin surface micromilieu (e.g., sebum and pH) between the two regions, since it is well-known that both sebum and skin microbiota can influence the immune functions of cells in their microenvironment (45–48). It is also important to keep in mind that differences in the composition of the sebum and microbiota seen between the two regions develop in an acquired manner during puberty. Naik et al. artificially established a microbiota change on mouse skin and observed that the induction of IL-17A is a relatively conserved response of the skin to an encounter with a new commensal, and these T cell responses were able to promote skin innate responses (production of S100A8, A9) (49). Since a similar, but physiological microbiota shift develops on the surface of human skin during puberty, we hypothesize that Th17/IL-17 immune milieu in SGR region could be the remnant of this SIS adaptation during puberty in SGR skin (Figure 5).

In the pathogenesis of immune-mediated inflammatory and autoimmune skin disorders SIS plays a crucial role. Some of these diseases favorably localize to special skin areas, such as acne, rosacea, and cutaneous lupus appear mostly on the face, scalp, and chest, which are SGR areas. Since until now the composition and activation of the SIS was considered unique on the whole body, other causes were investigated in the background of the region-specific localization of these diseases (sebum, microbiota, endocrine alterations, sunlight). Our present data allow to consider this question from a new aspect and raise the possibility that region-specific characteristics of SIS can have important commitment in the development of the region-specific immune-mediated skin diseases. The non-inflammatory Th17/IL-17 guided immune and barrier milieu of SGR skin probably predispose this area for the development of inflammatory Th17 type immune-mediated skin diseases, after disruption of steady-state condition, due to change in sebum, microbiota or sun exposure and endocrine status. Recent data from the literature support this hypothesis, since in acne, rosacea, and all forms of cutaneous lupus (DLE, SCLE, SLE), one of the major skin infiltrating lymphocyte subsets is the inflammatory type Th17 cell population (51–54). Our recent data also raise the possibility that disrupted tolerance and a switch from non-inflammatory to inflammatory Th17/IL17 milieu may have special role in the development of SGR localized inflammatory skin diseases, since in SGR skin during steady-state a homeostatic, probably tolerogenic TSLP epidermal expression was detected while a significant loss of this TSLP together with prominent influx of inflammatory DCs and inflammatory Th17/Th1 cells with IL-17/interferon-γ cytokine milieu were observed during the development of rosacea (7).

Taken together, our data call the attention to the proper selection of healthy skin controls in research and also to the development of new barrier restoring strategies that take into consideration the region-specific characteristics of the skin barrier and SIS.

## Ethics Statement

The study was approved by the local ethics committee of University of Debrecen, Hungary.

## Author Contributions

GB, ZD, TB, and AS designed the experimental protocol. GB, ZD, AK, and BM performed the experiments. KG and ZP collected the skin samples. ZH carried out the digitalization of immunohistochemistry slides. SP and ZD performed the analyses of RNA Seq data. GB, ZD, DT, TB, and AS interpreted the data. GB, ZD, TB, and AS wrote the manuscript. All the authors read and approved the final manuscript.

## Conflict of Interest Statement

The authors declare that the research was conducted in the absence of any commercial or financial relationships that could be construed as a potential conflict of interest.

## Abbreviations

AMP, antimicrobial peptide; BP, biological process; DC, dendritic cell; FA, field area; FC, fold change; GO, gene ontology; IHC, immunohistochemistry; Ig, immunoglobulin; IL, interleukin; ISP, immune system process; KC, keratinocyte; KEGG, Kyoto encyclopedia of genes and genomes; KRT, keratin; MA, Mask area; MF, molecular function; PCA, principal component analysis; ROI, region of interest; qRT-PCR, quantitative real-time PCR; SGP, sebaceous gland poor; SGR, sebaceous gland rich; SIS, skin immune system; TGF-β, transforming growth factor beta; Th, T helper; TLR, toll-like receptor; TNF-α, tumor necrosis factor alpha; TSLP, thymic stromal lymphopoietin.
