# Supplementary material for: Immunotopographical Differences of Human Skin
Source: Front Immunol. 2018 Mar 5;9:424. doi: 10.3389/fimmu.2018.00424 (PMC5844973; doi:10.3389/fimmu.2018.00424)
Supplement: Supplementary file 1 [file presentation_1.PDF]

Supplementary Material

Immunotopographical Differences of Human Skin

Gabriella Béke, Zsolt Dajnoki, Anikó Kapitány, Krisztián Gáspár, Barbara Medgyesi, Szilárd Póliska, Zoltán Hendrik, Zoltán Péter, Dániel Törőcsik, Tamás Bíró, and Andrea Szegedi*

*** Correspondence:** Andrea Szegedi: aszegedi@med.unideb.hu

Supplementary Figures

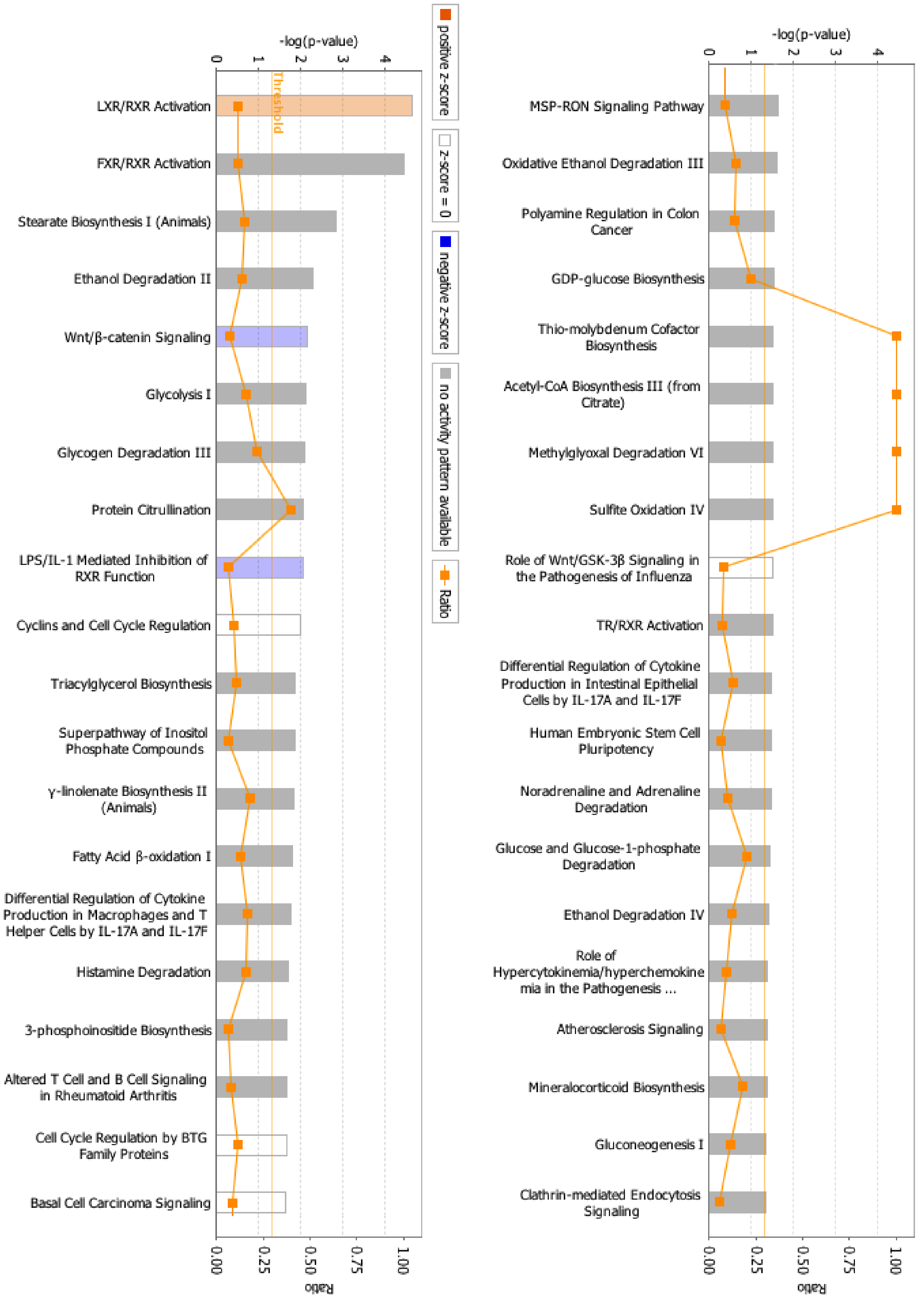

-log(p-value)
Threshold
LXR/RXR Activation
FXR/RXR Activation
Stearate Biosynthesis I (Animals)
Ethanol Degradation II
Wnt/β-catenin Signaling
Glycolysis I
Glycogen Degradation III
Protein Citrullination
LPS/IL-1 Mediated Inhibition of RXR Function
Cyclins and Cell Cycle Regulation
Triacylglycerol Biosynthesis
Superpathway of Inositol Phosphate Compounds
γ-linolenate Biosynthesis II (Animals)
Fatty Acid β-oxidation I
Differential Regulation of Cytokine Production in Macrophages and T Helper Cells by IL-17A and IL-17F
Histamine Degradation
3-phosphoinositide Biosynthesis
Altered T Cell and B Cell Signaling in Rheumatoid Arthritis
Cell Cycle Regulation by BTG Family Proteins
Basal Cell Carcinoma Signaling
Ratio
positive z-score
z-score = 0
negative z-score
no activity pattern available
Ratio
-log(p-value)
MSP-RON Signaling Pathway
Oxidative Ethanol Degradation III
Polyamine Regulation in Colon Cancer
GDP-glucose Biosynthesis
Thio-molybdenum Cofactor Biosynthesis
Acetyl-CoA Biosynthesis III (from Citrate)
Methylglyoxal Degradation VI
Sulfite Oxidation IV
Role of Wnt/GSK-3β Signaling in the Pathogenesis of Influenza
TR/RXR Activation
Differential Regulation of Cytokine Production in Intestinal Epithelial Cells by IL-17A and IL-17F
Human Embryonic Stem Cell Pluripotency
Noradrenaline and Adrenaline Degradation
Glucose and Glucose-1-phosphate Degradation
Ethanol Degradation IV
Role of Hypercytokinemia/hyperchemokinemia in the Pathogenesis ...
Atherosclerosis Signaling
Mineralocorticoid Biosynthesis
Gluconeogenesis I
Clathrin-mediated Endocytosis Signaling
Ratio

Supplementary Figure 1. Significantly enriched canonical pathways (FDR < 0.05) revealed by Ingenuity Pathway Analysis. Non-restricted Canonical Pathway Analysis revealed 40 significantly enriched terms (canonical pathway were automatically arranged by IPA based on the level of significance. Of these 40 canonical pathways, the first 14 in the significance ranking list were all related to lipid metabolism (such as LXR/RXR Activation, FXR/RXR Activation, Stearate Biosynthesis I, etc.). The first (i.e. the most significant) pathway exclusively related to the skin immune system was the IL-17 related one. Besides IL-17 signaling, the only pathway which could be partially connected to skin immune functions was "LPS/IL-1 mediated inhibition of RXR function".

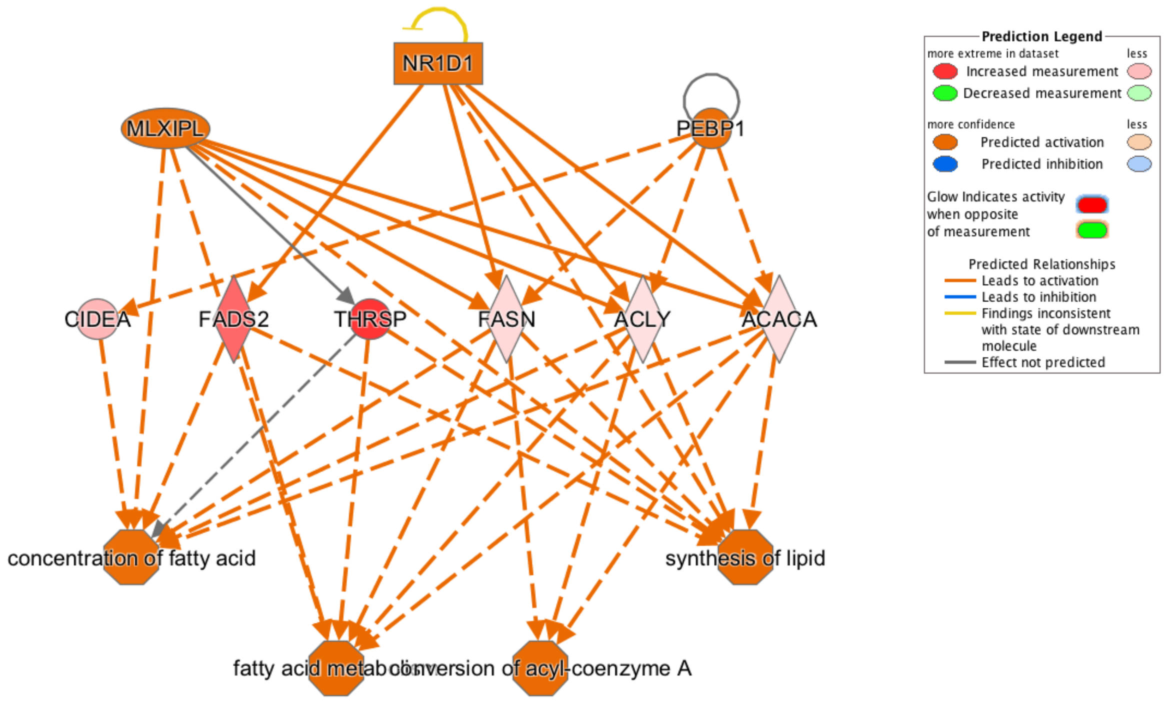

Supplementary Figure 2a

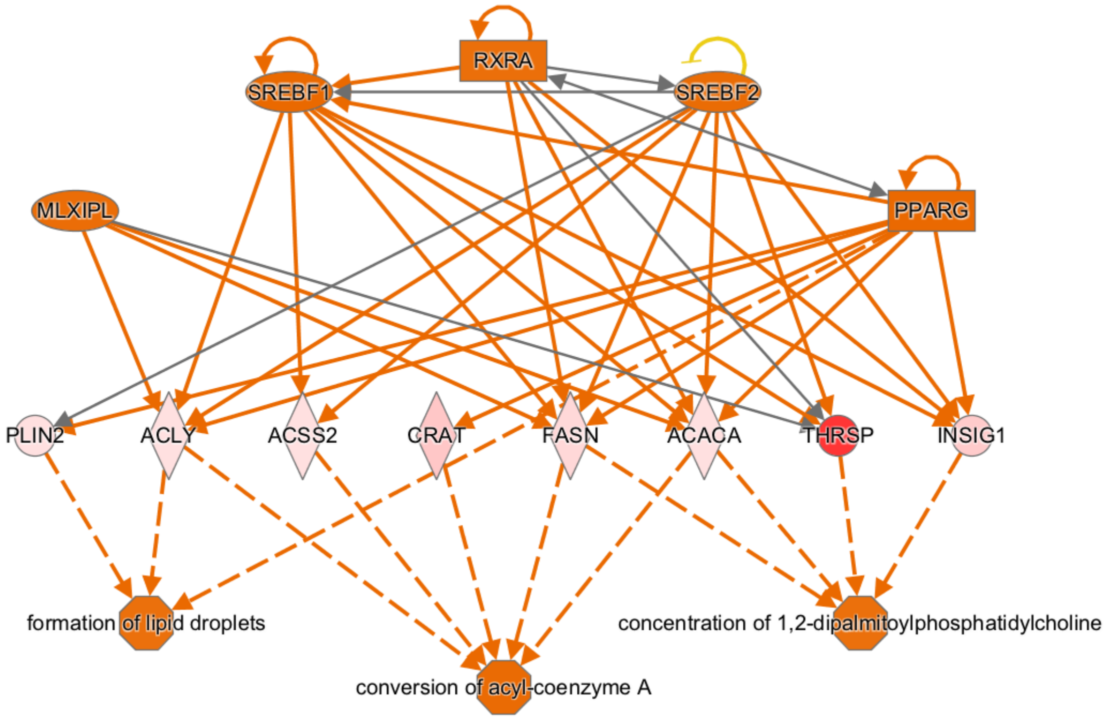

Supplementary Figure 2b

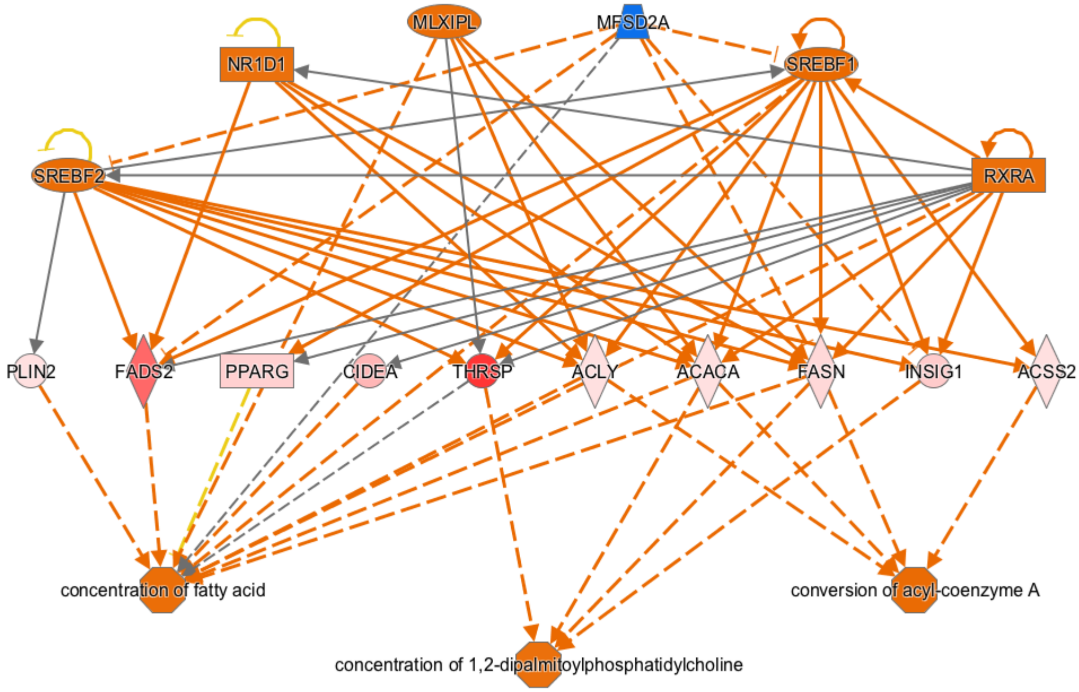

Supplementary Figure 2c

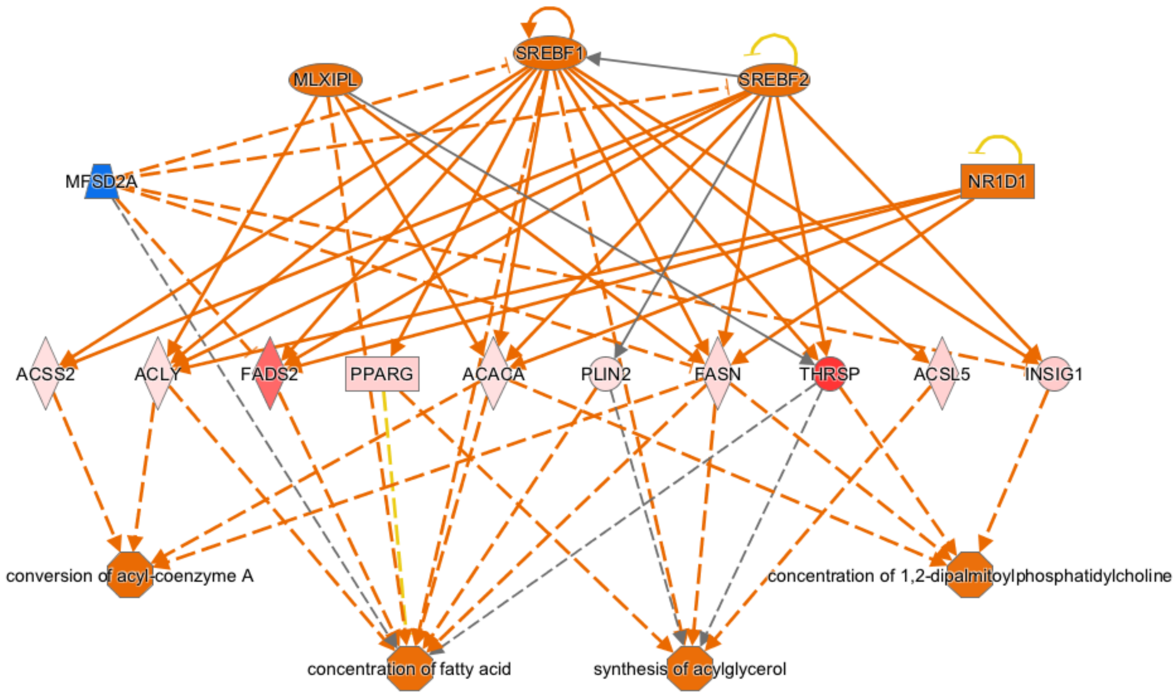

Supplementary Figure 2d

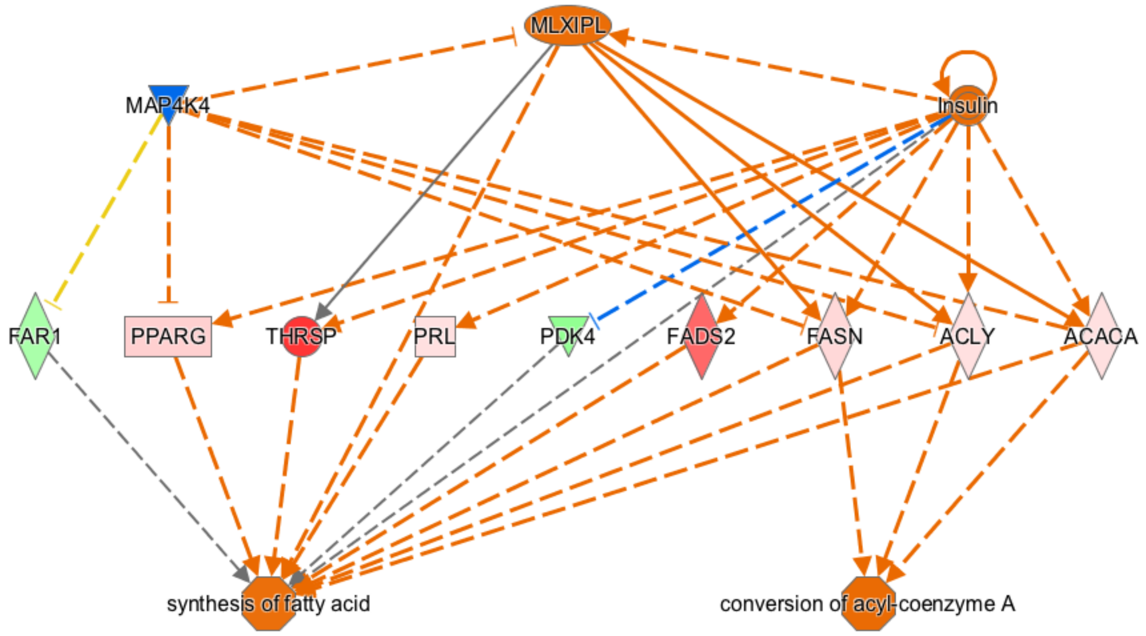

Supplementary Figure 2e

Supplementary Figure 2a-e. Cellular- and lipid metabolism-related Regulator Effect Networks revealed by Regulatory Ingenuity Pathway Analysis. Regulatory IPA Analysis revealed eight signaling networks in which both upstream regulators and downstream cellular responses were identified in relation to certain gene panels. Among these pathways five of these networks were linked to lipid metabolism.
